# Integrating a Lived Experience Position Into an Adult Survivors Program in Health: A Co‐Reflection of Lived Experience, Clinical and Academic Perspectives

**DOI:** 10.1111/hex.70891

**Published:** 2026-10-03

**Authors:** Charlene Hurwitz, Elizabeth May, Maya Hayden, Katie McCarthy, Sophie Isobel

**Affiliations:** ^1^ Northern Sydney Local Health District Sydney Australia; ^2^ The University of Sydney Sydney Australia

**Keywords:** adult survivors, health services, implementation, lived experience: child sexual abuse, peer work, reflection

## Abstract

**Background:**

Integrating lived experience roles into health services is increasingly recognised as key for effective service delivery. The Adult Survivors Program (ASP) has been established to support adult survivors of childhood sexual abuse, a population with complex health needs and historical mistrust of services. This paper explores the implementation of a dedicated Lived Experience position within ASP.

**Aim:**

To critically reflect on the process of establishing a Lived Experience of child sexual abuse role in a Violence, Abuse and Neglect health service, and identify lessons for workforce development.

**Methods:**

A dialogical co‐reflection approach was used, involving a Lived Experience worker, senior clinician, clinical coordinator, and two researchers. Over 6 months, fortnightly reflective sessions were conducted. Individual written reflections are presented to form a shared narrative of the experience of implementation.

**Results:**

The narrative highlights the transformative potential of Lived Experience roles in VAN, challenges of visibility and stigma, and the need for organisational readiness. Establishing trust, safety, and shared values was critical. Structural issues such as pay parity, scope clarity, and emotional load were identified as significant considerations. The Lived Experience role enhanced: survivor‐informed practice, disrupted hierarchical norms, and fostered cultural change, but also highlighted the importance of relational and systemic support.

**Conclusion:**

Embedding Lived Experience roles in health services offers opportunities for systemic reform and improved survivor engagement. Success depends on organisational commitment, flexible role design, reflective supervision, and sustainable resourcing. Findings inform policy and practice for integrating Lived Experience positions across health systems.

## 1 Introduction

Lived experience roles have emerged across health services, driven by rights‐based approaches and recognition of experiential knowledge as a distinct form of expertise alongside clinical and academic knowledge [1, 2]. Roles have been most well‐established in mental health settings where lived experience workers draw on personal experiences of distress, service use, and recovery to support individuals and contribute to service and systems change. In domestic, family and sexual violence services, lived expertise has long been informally embedded [3], with recognition of the unique experiences of lived experience workers in these services [4, 5], and recommendations for role establishment [6].

In research, partnership between conventional researchers and people with lived experience is also increasing, demonstrating a radical shift in how knowledge is produced [7]. This improves the quality and utility of research but is also an important matter of epistemic justice doing [7]. Positioning people and communities with lived experience as co‐creators of knowledge that holds power [8] challenges traditional paradigms of research and is crucial when seeking to find new ways to think and write about lived expertise. This paper uses a dialogical co‐reflection to produce lived experience‐led knowledge of the establishment of a lived experience of child sexual abuse role within a new team in a public health service.

The team is part of a statewide initiative in New South Wales (NSW), Australia known as the Adult Survivors Program (ASP). ASP was developed in response to the findings of a Royal Commission into Institutional Responses to Child Sexual Abuse. One of the key recommendations of the Royal Commission was that governments address existing sexual assault service gaps to provide advocacy and support and specialist therapeutic treatment for victims and survivors. This was based on recognition of the significant link between child sexual abuse and a range of adverse health impacts both in childhood and adulthood, including impacts on physical, psychological, social, and emotional wellbeing [9].

Many survivors may be reluctant to engage with health services due to historic harms, misuses of power or exclusion, as well as direct negative experiences of not getting their needs met [10]. ASP aims to support health districts to provide services for survivors of childhood sexual abuse with complex health needs, as well as building capacity of the health workforce who are supporting them. In one local health district in metropolitan NSW, a lived experience of child sexual abuse role was developed as part of establishing the ASP. This paper aims to present the dialogical reflections of the Lived Experience worker, clinical colleagues and academic collaborators on the role establishment.

The language used in talking about Lived Experience roles is important. Across settings the term Lived Experience is used broadly to refer to diverse experiences relevant to contexts. In this paper capitalised ‘Lived Experience’ is used to refer specifically to Lived Experiences of child sexual abuse. At other times, the broader uncapitalised term ‘lived experiences’ is used to refer to diverse experiences that people carry in their lives and work, which may include child sexual abuse or other types of violence, abuse and neglect.

## 2 Methods

### 2.1 Setting

Within public health services in NSW, VAN services provide specialist responses, including crisis, medical, forensic and psychosocial support to people impacted by domestic, family and sexual violence or child abuse and neglect. While VAN services focus on responding to violence, abuse and neglect presentations, ASP is focused on system‐wide coordination to strengthen coordinated responses across health services for adult survivors of child sexual abuse who also have other intersecting health needs.

ASP in the local setting commenced in 2024 with employment of a temporary program manager recruited to support program establishment and a permanent clinical coordinator. Further funding was sourced to employ a senior clinician and a Lived Experience worker who commenced in 2025, managed by the clinical coordinator. Two researchers from a nearby university were engaged to support evaluation.

The ASP Lived Experience position requires the worker to draw on their own lived experience of childhood sexual abuse and interactions with the health system. As part of program establishment, the position was designed to trial Lived Experience initiatives within ASP and support co‐design of the service. The co‐design elements of the program establishment are being written up by the co‐design group and will be published separately.

### 2.2 Design

This reflective paper uses co‐reflections to explore the process of establishing the Lived Experience role within the team. Herein, ‘the team’ refers to the Lived Experience worker, the senior clinician and the clinical coordinator. A process of critical reflection was undertaken to establish the basis for the co‐reflections. Critical reflection throughout initiatives helps build intentionality, sustain cultures of learning and adapting which then allow initiatives to be transformative [10]. Reflection on practice looks inwards, but can also be used to contribute to knowledge, through a ‘passing on’ of professional knowledge learnt in other ways to formal research [11]. Critical reflection in this project was facilitated by the two external researchers, contracted by the health service to document team learnings. As reflection occurred within existing roles, included no data collection, and was co‐authored by all engaged individuals, ethics approval was not required.

From March to August 2025, the team met regularly with the researchers individually or as a group. Reflective sessions were loosely structured around events and experiences, with opportunities to consider thoughts and feelings related to the work. Regular reflective sessions are known to be beneficial in enabling open conversations around the impact of power dynamics in processes where lived expertise and clinical expertise intersect [12]. Reflection in healthcare enables thinking critically about choices and actions and understanding the broader contexts within which these choices are made. Reflection can also enable ‘radical critique’ of hierarchies of knowledge [13]. Thus, the conversations served a purpose of protecting time to think about the work, holding of practice wisdom within the team, and trust building with the researchers. Trust in work with survivors takes time, and this was modelled in the process of establishing the research [14].

At the conclusion of the reflection period, it was planned that the researchers would develop themes from the transcripts. Themes included that knowledge from other fields isn't directly translatable to ASP, organisational readiness is important and contextual, there is pressure and possibility in the need to do something, centring survivor needs requires time and reflection, support for staff is essential, and measures of success need to be flexible. While these themes were reflective of the experiences of the team overall, distiling experiences into shared themes also resulted in a loss of the variation across narratives and reduced a process filled with contradiction and change down to its substantive elements. Ontologically, narratives present one part of reality, among many others [15] and the importance of the individual narratives only became apparent when they were removed in the process of theming. Thus, a structured reflective exercise was undertaken by each member of the team who responded to series of prompts about their experiences of integrating lived experience positions into health services (see Table 1). The prompts were broad and developed by the researchers based on conversations about what was ‘lost’ in the theming, including the changes that occurred over time and the experience of the team within the wider service context. The prompts were used by each individual to document their reflections.

**Table 1:** Prompts for reflections.

Prompts for reflections
How did you imagine the Lived Experience role would fit in ASP, when you first became aware of it?
How has your thinking changed since the beginning around how Lived Experience roles fit into VAN services?
Looking back now over the last year, what have been the most significant learnings for you about Lived Experience roles in the adult survivor space?
What have you experienced as helping you build a team of clinician and Lived Experience workers?
What do you think is unique about Lived Experience roles in VAN services?
What potential do you imagine for Lived Experience roles in VAN spaces in health services?

After each person had written their reflections, they were discussed as a group. A process of collaborative editing was undertaken to organise and refine reflections. While everyone had the opportunity to refine their words, the essence of each reflection was maintained to support the ‘sincerity’ of the dialogical approach [16]. The process of writing the paper was led by the researchers, with a sustained dialogue amongst the team about any changes made. Following collective reading, refinement and discussion, excerpts of the reflections are presented as they were written, organised under collated heading topics, while holding the individual story of each, to form a textual conversation. To support trustworthiness and reflexivity, each participant in the dialogue also provided a positionality statement about the perspectives they bring to the work.

#### 2.2.1 Charlene (Lived Experience Worker)

I bring the combined lens of a victim‐survivor of CSA, mental health peer worker, and social worker; a perspective shaped by navigating both sides of the system and the emotional terrain in between, an identity hard to separate. I began my career as a community support worker within a non‐government organisation supporting people with complex mental health needs. My insight from walking alongside people in community, and from my own lived experience, deepened my belief that recovery is not a service delivered. It's a relationship that grows from humility, shared humanity, and authentic partnership. I regard lived experiences as professional knowledge, formed through reflection, integrity, and purpose, and deeply influenced by the moral injury that occurs when systems silence personal truth or obstruct ethical action. I have both experienced and witnessed the harm caused by systems that silence or pathologise survivors. I have also seen the healing that begins when people are met with respect and genuine hope for change. As a peer, I build my practice on mutual learning and relational ethics, guided by Intentional Peer Support. I also draw on anti‐oppressive and trauma‐informed approaches, shaped by my own and others’ experiences of the health system.

#### 2.2.2 Maya (Senior Clinician)

I came to the adult survivor role as an experienced sexual assault counsellor. I had worked supporting Adult Survivors through counselling and court support and prior to that, held Social Work roles within primary and community health with adults with complex comorbidities and acute domestic violence presentations. My practice is informed by my interest in the social constructions and intersections within societies that cause harm to individuals. I have interest in the legal system and justice theory as Adult survivors have often experienced multiple forms of harm and systemic barriers that prevented them to access safety or be supported post‐harm. I also have a close family member who was sexually abused as a child, and I was the first person they disclosed to in adulthood. This has shaped my professional career and how I consider the whole picture of a survivor's life.

#### 2.2.3 Liz (Clinical Coordinator)

I am a social worker and have worked in clinical roles in the grief and loss and VAN sector for the past 16 years. I have predominantly worked in sexual assault or domestic violence, as well as policy. In my time in violence against women services, I have watched survivor advocates fighting for change across the system and have watched how these roles have started to be incorporated within systems. This change has been significant to witness and has also been interesting to watch as both a clinician and a person with my own lived experiences within the VAN space. Over the course of my career, I have reckoned with what my own experiences mean for my practice and the practice of other clinicians with lived experiences. I have always chosen roles that supported and allowed me to work in ways that align with my personal and professional values including core social work values, feminist frameworks, anti‐oppressive practice and trauma and violence informed work, Working within the ASP has been an opportunity to work in an area I feel passionate about, and to use my practice knowledge to support systemic change.

#### 2.2.4 Katie (Research Officer)

I am a social worker and lawyer by background. I have worked as a social worker in domestic violence services in remote areas and in health. At the commencement of this work, the social and emotional wellbeing model informed my thinking about survivor‐healing and the connection between the survivor, their communities, systems and their wider environment. A feminist and trauma‐informed approach also inform my thinking about how to navigate safety, trust and power. I do not declare any lived expertise in the work.

#### 2.2.5 Sophie (Senior Researcher)

I am an academic and a mental health nurse. I have worked for many years to improve mental health care delivery and in more recent years, researching trauma informed care through critical methodologies. I always try to work in trauma and violence informed ways. That is, acknowledging the impacts of adversity on people's lives, while also acknowledging the ways that systems and services can reinforce trauma through structural violence. I came to the Adult Survivor work committed to being a part of work that tries to improve the experiences of folks accessing health services in the context of lifetime trauma. I liked that the team were wanting to engage in critical reflection on the process, not just focusing on the outcomes. My experience has been that reorienting health services to be sensitive and responsive to trauma is slow and challenging work and I was interested in the opportunity to actively document this process. My work in trauma informed approaches is informed by my own experiences of childhood and lifetime trauma, but I am conscious that while this informs everything I do, it isn't the professional space I occupy.

## 3 Findings

### 3.1 Imagining a Lived Experience Role in an Adult Survivors Program

#### 3.1.1 Charlene (Lived Experience Worker)

When I joined the ASP, I carried frustration, pain, and hope. I wanted to help build a safe and sustainable peer workforce within VAN that deepens understandings of trauma and brings more compassion and safety to survivors. I felt strongly about supporting staff like me, who carry histories of child sexual abuse and complex trauma. I understand the challenge of holding these experiences while also caring for others in systems that can overlook them. I hoped this program and funding would create lasting change and help prevent the harm that survivors have experienced within the health system. I had concerns about representing survivors well enough. Was I ready to speak publicly about my own story, when I'd kept so much private? I was stepping into a new space of advocacy and visibility, and that felt daunting. The uncertainty made it hard to know what stepping into leadership as a survivor advocate would feel like, or the pressure I'd place on myself to represent both VAN and peer perspectives well. Transitioning from a clinical to a lived experience role reminded me of the persistent stigma surrounding lived experience work. Comments questioning my choice and career direction highlighted how deeply the system still misunderstands the skill, value, and expertise lived experience brings to care. My role is to speak truth to power, and it's taken time and reflection to learn how to do that safely, in ways that can positively influence the system.

#### 3.1.2 Maya (Senior Clinician)

I initially had a lot of trouble imaging what the Lived Experience role would be like. I was interested to have a lived experience colleague but wasn't sure what the role would do in practice, especially without any direct client facing work initially. I also had some concerns about how our disciplines would interact in terms of responsibilities: How would we deal with differences in responsibilities within shared work such as different priorities, documentation requirements or scopes of practice? What I was excited for, however, was to have real survivor voices. I felt that this would help decisions in the program be taken seriously and validate the recommendations of the program.

#### 3.1.3 Liz (Clinical Coordinator)

I was enthusiastic about the Lived Experience position in the ASP and have worked hard to establish and understand the opportunities of the role. I had hopes and excitement, alongside some wonderings and worries. I have always placed high value on lived expertise, however felt wary of introducing specific roles. I have never worked in services where peer workers are common, nor have I worked with a designated Lived Experience role before. I wanted to interrogate my own assumptions, understand how these roles function in other services and think about what was needed for the VAN space. I am cognisant that in VAN such a high proportion of the workforce do have a variety of lived experiences and often do not feel safe to disclose, and I was keen to think about what this introduction meant for the existing workforce who may have their own lived experiences.

#### 3.1.4 Katie (Research Officer)

I saw the Lived Experience role as an exciting opportunity to hear the voices of survivors, and for their experiences to make much needed change to health systems. I hoped the Lived Experience worker's knowledge, experience and perspective would be at the core of ASP. I was apprehensive about whether the Lived Experience role could be valued and honoured by the health system, which often does not meet the needs of survivors. I also worried about the enormity of the role – one person navigating the new space of using their lived experience knowledge to try to influence and bring change to the health system which has quite entrenched cultural norms and ways of working, and in which survivors have experienced harm.

#### 3.1.5 Sophie (Senior Researcher)

I have done a lot of work with Lived Experience colleagues in mental health, child protection, perinatal, and domestic and family violence which informed my assumptions of how the ASP Lived Experience role may work. I brought hope and worries. I have observed that Lived Experience roles always bring depth of perspective and opportunities to think differently about the work. I was hopeful to see how it could work within the ASP space and felt optimistic based on the enthusiasm and commitment of the team to setting up the role in a meaningful way. Initially I worried about things I have seen happen before where single lived experience positions can become a lone voice of change against a big system and as Charlene was previously a clinician, I was unsure about the paradigm shift required to go from being a clinician to being a peer within the same system.

### 3.2 Learnings About Establishing Lived Experience Roles in Health Services

#### 3.2.1 Charlene (Lived Experience Worker)

I hadn't expected this role to prompt so many personal disclosures from family, friends, colleagues, and people in my community. It was confronting to see how much harm remains unspoken. When other workers across health began sharing their own experiences of abuse, I was reminded of how deeply this issue runs through our workforce, shaping our collective practice, wellbeing, and workplace culture.

I have learned how important it is to name and align personal and professional values early. This work relies on trust, which takes time, a luxury not always available within tight project timelines. What I have come to appreciate is that there's an ever‐present tension across the health system between reaching more people, or providing more comprehensive and relational care to each person. Both are essential, yet the system is not adequately funded and resourced to do both.

This role has been the hardest and most rewarding work I have ever done. It stretches me daily, personally and professionally. I have learned so much about myself and have grown in confidence as a survivor advocate. I have learned that systemic advocacy is slow and resource intensive. Change takes time and persistence. I have also learned that this role demands emotional agility, the ability to sit with discomfort, navigate competing truths, and hold systemic hope within bureaucracy and workforce constraint.

#### 3.2.2 Maya (Senior Clinician)

Working with Charlene has been a great experience of mutual learning. It took time to find the rhythm and interaction between our roles as we both navigate roles that are not always clearly defined, but sharing thoughts and exploring ideas has been very powerful. My worries about how our roles would work together were unfounded as it has felt validating to see that the Lived Experience perspective matches a lot of the clinical perspective, but through our work together I have also gained insight into systemic processes that could be improved for survivors both working within and accessing the system. It sometimes feels like an ebb and flow between excitement and frustration – usually around bureaucracy. A challenge I have found is elevating the Lived Experience voice when Charlene is only one person; it feels like a lot of pressure on one person. Lived Experience roles should be embedded in decision‐making and policy and procedure. Charlene has also helped me understand a lot about hierarchical challenges for survivors within settings where they can be pathologised. It highlights to me a bigger need than solely capacity building within these systems, but advocacy for more adequate resourcing and better relationships between services, to improve outcomes for survivors.

#### 3.2.3 Liz (Clinical Coordinator)

So much has developed in my thinking since joining this team and recruiting and implementing the Lived Experience role. I have found the inclusion of a dedicated Lived Experience role invaluable for the program. The learning never stops, I am constantly thinking and re‐thinking ways we can shape the role moving forward to ensure it maintains equity within the team, that the scope meets the needs of the program in ethical ways that reflect the value of lived expertise and how to do that within a system where this type of work is still so new. I think a challenge is that we often want to ‘get it right’ and yet because the learning is so new I know that I am not always ‘getting it right’. I feel lucky to have a team that gives me grace. Another challenge is the time that things take, there feels like there could be more time for the thinking, reflecting and integrating that needs to happen to lay the foundations for more roles in the future.

#### 3.2.4 Katie (Research Officer)

It has been incredible to observe what Charlene has achieved in establishing the role through bringing survivor voices in where they weren't previously, co‐design, holding space, networking, advocacy, and optimism. The co‐reflection provided a space to share experiences, perspectives and knowledge, both personal and professional. This coming together, being honest and vulnerable together, has really helped me to understand the meaning of the Lived Experience work, the value of lived expertise, and the challenges of this work in the health system. This listening, sometimes sitting with discomfort, bringing to light what is often silenced, and re‐learning ways of knowing and doing is important in making change.

#### 3.2.5 Sophie (Senior Researcher)

Over the time that we have known the team, the incredible opportunities that a voice of Lived Experience can bring to this work has become apparent. Personally, I have become much more conscious of the differences of Lived Experience roles across settings. At times they get professionally grouped together as a peer workforce, but the challenges are also unique to the experiences and communities. I have become more aware of how the stigma associated with Lived Experience roles across settings can also mirror the stigmas that the unique communities face. For example, the mental health lived experience workforce seem to get risk and vulnerability weaponised against them, the child protection lived experience workforce have to navigate power and coercion associated with past and present parenting, and I now see how lived experience roles in adult survivor spaces may experience exclusion, othering and shame in ways that align to survivors’ experiences more widely. As a researcher I have been conscious of the need to be trauma informed in the process of research and have had to think differently about how to evaluate and write about lived experience work.

### 3.3 Navigating Power, Safety and Trust in a Team of Clinician and Lived Experience Workers

#### 3.3.1 Charlene (Lived Experience Worker)

Moving from a clinical to peer role has shown me that accountability looks very different depending on where you stand, even within a closely aligned team. Clinicians are accountable to organisational policy and clinical frameworks, while peers will always hold accountability to the community and survivor truth. The tension between the two is uncomfortable but it's what challenges systems to remain human, and prioritise what matters. As a team we have remained open and honest about our priorities and pace of innovation, all aware of the personal cost to survivors if this work isn't approached with deep reflection and careful consideration, something we've had to continually acknowledge and negotiate together. Tensions have surfaced around the scope of my role, how best to facilitate co‐design opportunities, how to manage the pace of innovation and balancing representation opportunities. Managerial support to help focus my priorities and direct my efforts has been essential, especially when everything has felt important to me. At times when I've been stretched too thin or feared my contributions might become tokenistic, we've had honest and sometimes difficult conversations that have helped me refocus.

Being a ‘first’ in a system brings both privilege and vulnerability. Visibility has challenged stigma but can also invite scrutiny. I've felt very supported to utilise my lived expertise, yet also a self‐imposed pressure to represent well, speak for others, and embody Lived Experience leadership in a space that is deeply personal. I hadn't expected to rely so deeply on my community for grounding and encouragement. Their support and shared understanding helps me stay centred and continue this work through both personal and professional challenges. I have had to navigate difficult conversations with loved ones and my community about taking this role and seek consent to share a story intertwined with theirs. This process has strengthened my relationships and helped me address past hurts/silence that I would not otherwise have addressed. I've moved further in my own recovery journey through doing this work, inspired by the courage of other survivors to speak boldly and authentically.

#### 3.3.2 Maya (Senior Clinician)

I think the start of building ‘safety’ between the clinical role and Lived Experience role has simply come down to getting to know each other, not just on a professional level. Our relationship does not revolve around just our work in ASP, but also a friendship. We have been supportive of each other and whilst our roles are important, our family and health are more important than our roles. We talk about the differences of our professional backgrounds – for example how we both have historically measured risk or how we approach processes. We do not have to agree as a team, but there is mutual respect and freedom to speak from our different perspectives. The ASP team is unique to any other team I have worked in because sitting and talking about ideas has been a key part of the work.

#### 3.3.3 Liz (Clinical Coordinator)

I think some of the early training and research I did about my role in managing lived experience roles fundamentally changed my understanding of lived experience as a discipline. This has laid the foundation of the role in ASP and how I manage and support the role. I haven't line‐managed a designated lived experience position before, but it has changed my practice around the types of support, conversations and advocacy required for people employed to draw on their lived expertise while also employed within the system. While it's not a wildly different way of being to how I am in my role as a manager of a team of clinicians, there are nuances of needs. As it is a new discipline it is important as a manager not to be relying on your worker to teach and upskill you in their discipline and I am working in an ongoing way to attune to the needs of these different workforces as part of my ongoing practice.

The way we have been able to establish the roles as equal has really paid off in terms of power, safety and trust. The only exception to this is pay. Mirroring funding, seniority and contribution to the team between clinical and Lived Experience roles in the ASP moving forward is important for the structure of such a small team and to the success of the Lived Experience role. So much has gone well; so many of the wonderings and hesitations I held prior to recruiting, didn't seem so big once Charlene started.

#### 3.3.4 Katie (Research Officer)

From the perspective of sitting outside ASP, things that seem to have been helpful included having Charlene sit on recruitment panels for key roles, and mandatory organisational readiness training for all VAN staff prior to her commencing. Ongoing learning and training opportunities have seemed important for staff to critically reflect and understand the value of Lived Experience roles. It has taken time to build safety and trust in the process of engaging with the team for research. This has required commitment, critical reflection and vulnerability from everyone involved. Through this we have built trust, listened deeply, persevered, and learned from each other.

#### 3.3.5 Sophie (Senior Researcher)

We were privy to some of the planning before Charlene started in her role, and the thought that went into trying to juggle flexibility of how she may want the role to be, and the need for structural guidance to reduce burden upon starting. We have witnessed thoughtful attention to the days the team would work, where they would sit, and how often they would meet, to ensure collegiality and trust. In our research relationship there have been some challenges, but the team have been open to ongoing collaborative reflection, not least evidenced by their engagement of us as researchers to observe and document their experiences. This is demonstrative of a curiosity and willingness to understand and adapt which has required us as researchers to remain conscious of power and trustworthiness in how we observe and write about experiences that are not our own.

### 3.4 The Possibilities for Lived Experience Workforce Within Van Services

#### 3.4.1 Charlene (Lived Experience Worker)

Like any role designed to drive cultural reform, it is true value lies in system translation, bridging survivor wisdom with the operational and policy frameworks that shape care. This work cannot be done alone; it relies on a supportive clinical team and adequate resources. While forms of violence, abuse, and neglect often overlap, I believe child sexual abuse specific lived experience roles are important to maintain, because they hold distinct knowledge of childhood sexualised trauma, complex relational lifelong impacts, and unique barriers to disclosure and help‐seeking. At the same time, it is exciting to see the potential for VAN to grow lived experience roles more broadly.

VAN is well positioned for a lived experience workforce, but workers will need to be supported with protected time to meaningfully engage with community, build relationships, and learn from others with shared experience as victim‐survivors and peer workers. When developing position descriptions and individual key performance indicators, flexible work arrangements must be thoughtfully considered and built‐in to support sustainable practice, given the demanding and emotionally complex nature of the work. Managers and teams must recognise the emotional load of lived experience work and provide reflective, relational supervision that balances support with accountability and transparency. Shared understanding should be communicated and respected from different perspectives to maintain alignment, build trust, and protect against lateral and structural violence. Without clear boundaries, lived experience workers can be stretched across competing priorities, increasing the risk of burnout.

Peer work to me feels like a personal protest. It gives me what the system often takes away: space to be human, to respond with empathy and flexibility. It lets me use what I have learnt, both clinically and through Lived Experience, to stand beside other lived experience workers and protect the integrity of our growing workforce. What I once saw as personal sensitivity, is better reframed as ethical sensitivity. It reflects deep awareness of risk, harm, and power shaped by lived expertise.

#### 3.4.2 Maya (Senior Clinician)

I think VAN would benefit from multiple Lived Experience roles to reduce isolation and to embed survivor voices into our practice and processes. But we must think carefully about the impacts on people whose voices we're trying to elevate within our services and the repercussions when these are not heard or incorporated into systems or processes. I experience this system fatigue as a clinician, but I think, when it is also a deeply personal experience embedded in your role, it hits harder.

I also understand that across peer and clinical workforces there are varying opinions of the term ‘lived experience’ and whether a lived experience role would encompass broader violence, abuse and neglect or more specific life experiences relevant to the role (e.g. child sexual abuse). I personally have found the more specific ASP Lived Experience role invaluable, as although trauma and experiences are always going to be varied across individuals, there are dynamics and particular elements to certain forms of abuse that are more deeply understood from one survivor to another.

#### 3.4.3 Liz (Clinical Coordinator)

Something unique about Lived Experience in VAN roles, is that VAN services have evolved from survivor‐led services of the feminist movement. The principles of VAN services and peer work are very closely aligned. For our team ‘Lived Experience’ is considered Lived Experience of CSA, but I have been trying to imagine the future for lived experience VAN roles and the different ways these could be structured. There is so much potential for lived experience work in VAN services across policy, education and training, leadership and frontline facing roles.

#### 3.4.4 Katie (Research Officer)

VAN services generally have a good understanding of power, control and abuse compared to other parts of the health system and include therapeutic approaches to healing. They therefore seem well placed to create space for lived experience roles, but wider systems issues will still impact. Lived experience workers can bring so much to health, and it is important to make changes in wider health systems to ensure workers are supported.

#### 3.4.5 Sophie (Senior Researcher)

Observing the beginnings of Lived Experience identified roles within VAN is a privilege. It is a space with so many existing structures and values that can support the workforce to define its scope and ways of working. There will be some ongoing shuffling in the wider VAN workforce, which is staffed by people with deep commitments to survivor advocacy, to find a way to make space for expertise gained from declared lived experience, alongside clinical expertise. It may also lead clinicians to become more conscious of their own paradigms, including the ways these may commonly be shaped by undeclared lived experience. Lived experience roles have the opportunity to destigmatise violence, abuse and neglect experiences, but also require disclosing elements of life that are still stigmatised within services. The lived experience workforce seems to be being asked to publicly and professionally declare experiences to change systems, with recognition that those systems are not yet set up to address their needs or value this expertise.

## 4 Discussion

This paper used dialogical narratives to explore the implementation of a Lived Experience position into an Adult Survivors Program, within a health service. Considering how to evaluate or document the establishment of lived experience roles requires innovative approaches [17]. As the work of peer work is relational and transformational, methods of evaluation should align to the ontological positioning of lived expertise as knowledge. The experiences and perspectives of the team explore the challenges and possibilities for lived experience positions across health, contributing new knowledge on implementation processes and considerations. The findings reflect unique elements of the local context, but they also contribute to practice‐based evidence to inform wider systems work to establish lived experience workforces. Following the reflections, the team collaboratively constructed a list of recommendations based on the ‘lessons learned’ (see Table 2). Many of these recommendations align to existing best practice frameworks for embedding lived expertise advocacy across services [2, 5, 6], while also identifying particular elements for lived experience roles embedded within VAN contexts in mainstream health services.

**Table 2:** considerations for establishing lived experience roles with violence, abuse and neglect services in health.

**Organisational readiness and governance**	Secure visible senior leadership commitment and resourcing
	Establish formal governance inclusion of lived experience roles in recruitment, policy and planning
	Build in organisational readiness training
	Embed reflective evaluation processes
**Role design, scope and safety**	Draft clear but flexible position descriptions that name specific lived expertise matched to the program
	Articulate differing responsibilities of clinical and lived experience roles in client care
	Keep scope flexible and balance creativity with defined responsibilities to avoid tokenism and burnout
	Include protected time for survivor/community engagement
	Align KPIs with survivor‑driven outcomes
	Consider the name of roles and how they reflect specific lived expertise
**Recruitment and onboarding**	Use inclusive recruitment that values lived expertise, include lived experience workers on panels
	Acknowledge visibility impacts of public identification and support staff transition into the role, acknowledging the vulnerability involved
	Onboard with explicit discussion of power/safety/trust and role boundaries and escalation pathways for safety or workplace concerns
	Resource external peer networks, reflective supervision, and guard against lateral and structural violence
	Normalise the emotional load and build fair adjustments/flexibility into conditions
	Consider how leave arrangements can reflect the known health challenges experienced by people with lived experience of violence, abuse or neglect
**Team integration and culture change**	Educate teams on peer practice values
	Use co‑production to transfer power, reduce stigma, and embed Lived Experience in decision‑making
	Schedule structured reflective sessions to surface tensions, negotiate accountabilities (community vs organisational), and build trust
	Facilitate inter‐team opportunities to ensure integration of knowledge across service
**Pay parity and career pathways**	Commit to pay parity, transparent grading, and progression pathways that value lived expertise equally with clinical expertise
	Avoid lone roles (or where possible create links to partner workforces and community) and create leadership pathways
**Evaluation and sustainability**	Build practice‑based evidence using dialogical reflection and co‐design
	Establish mechanisms of safe resolution of difference of opinions

The team was established within a supportive service, built upon values of survivor advocacy and committed to thoughtful and careful implementation. Ensuring a supportive organisational and team context for lived experience roles is known to be essential for minimising the known challenges [18]. However, establishing lived experience roles in any new space will still bring obstacles. The team continue to grapple with defining lived experience, as well as issues associated with visibility, paradigm clashes, and stigma within the wider workforce. While lived experience roles are set up to disrupt these things, as new additions to the workforce, they seemingly also encounter them. The introduction of lived experience roles challenges traditional hierarchy of healthcare systems [19], activating role tensions and resistance. While the current team were able to manage these tensions through relational safety and trust, any implementation requires built in time and support to manage the disruption that should be anticipated as part of the process.

The reflections highlighted the nuances of lived expertise and what this refers to. ‘Lived experience’ is a broad term associated to a discipline or practice, as well as people’ s diverse experiences within it. The importance of how lived experience roles are named and defined has been recognised where roles are more established [1]. Role titles are important for maintaining the distinctness of lived expertise, but how specific to be in defining the nature of the experiences being drawn upon is not always clear [20]. In Australia, national guidelines [21] identify that what events or experiences lived expertise relates to is less important than the knowledge, skills and wisdom gained. Yet there is a risk of assuming sameness when lived experiences of violence, abuse or neglect, as well as collective recognition and responses to them, are all very different. In ASP, a specific lived experience of child sexual abuse was identified as crucial for community engagement. However, this raises difficulties, as workers should not have to disclose all elements of their experiences. Being in an identified lived experience role is linked to experiences of workplace stigma and emotional labour [22], with additional personal impacts apparent when the identified ‘experience’ also impacts families and communities. Clear naming of roles, description, scope and expectations are important to ensure people are employed to safely advocate for communities to which they are connected or embedded, however ongoing consideration of how to support people with this visibility is required.

Across settings, many lived experience roles are a single worker within a wider team, established as a pilot initiative or needing evaluation to justify ongoing funding or additional positions [23]. Subsequently many workers are required to establish, perform, and evaluate their roles to justify their value. Yet despite these expectations, payrates for lived experience workers are low compared to other health professionals [23, 24, 25]. In ASP, pay for the Lived Experience position was negotiated based on equity across expertise and a co‐valuing of clinical and lived expertise, but this isn't always possible. It is an ongoing challenge for the wider health system to work towards fair and reasonable pay and conditions for people being asked to draw on their life experiences daily in their work [23, 24, 25]. While lived experience is increasingly recognised and valued across settings, resourcing and continuing positions are crucial for ensuring sustainability of the workforce [26].

Lived experience work is both professional and personal. While all team members developed their thinking and understanding of Lived Experience work over time, personal impacts were directly expressed by the Lived Experience worker. The growing literature on lived experience work highlights both its transformative potential and its inherent challenges. Personal impacts including purpose, empowerment, identity development, and satisfaction, alongside emotional labour, exhaustion and retraumatisation [23, 27]. Employing people with lived experience into the same systems from which they have historically received inadequate support [27] and expecting individuals to be the lone voices against exclusionary policies, processes, and cultures, can have implications [23]. The reflection adds detail to this understanding, suggesting how the impacts of the response of systems to lived experience roles can reflect the experiences of the wider survivor community, as well as the personal impacts of being in identified roles named based on experiences. Even within supportive teams there is risk of lateral or structural violence for lived experience workers, without ongoing systemic change. In ASP, alongside professional growth, unexpected challenges arose from collegial disclosures, changing relationships with the wider survivor community, and the effects of publicly declaring lived experience of child sexual abuse. However, benefits resounded when trust and relational safety were established, in line with models of trauma recovery and the known importance of connection to community, justice and meaning‐making [28].

## 5 Limitations

There is an imbalance of voices in this narrative based on the reflections of one Lived Experience worker, two clinicians and two academics. The paper also does not include the perspectives of people accessing ASP. The Lived Experience worker had a social work background which may have influenced trust, safety and managerial practices within the team. Dual professional identities can allow for differing privileges and may reduce the risk of perceived liminality or existing ‘between two worlds’ [29, 30], however a reverse stigma can also occur where legitimacy of lived expertise is challenged. When lived experience roles are managed within clinical teams, challenges commonly occur [31], The shared professional and personal backgrounds of the ASP team may have lessened this.

## 6 Conclusion

The implementation of lived experience roles across health services, particularly for communities who have been historically excluded or harmed in care, is an important step towards redistributing power, supporting transformational change, and improving health outcomes for individuals and communities. As positions are set up across health settings, there is a need to collaboratively explore and document the lessons learned, to ensure a building of knowledge over time and to reduce burden upon individual workers and teams to have to ‘figure it out’ as they go.

This paper used a dialogical narrative storytelling method to capture the process of implementing a Lived Experience position in a new team supporting health services to respond to the needs of adult survivors of childhood sexual abuse. This approach sought to hold the varying stories of each perspective in their wholeness, rather than merging, synthesising or genericising them through a collective analysis, while still seeing each as a part of a whole. The experiences of the team explored changes in perceptions over time, lessons learned about the importance of trust, safety and support, and considerations for teams establishing new lived experience positions across settings.

## Author Contributions

**Charlene Hurwitz:** conceptualisation, data curation, writing – review and editing, investigation, formal analysis, methodology. **Elizabeth May:** conceptualisation, methodology, data curation, investigation, formal analysis, writing – review and editing. **Maya Hayden:** investigation, data curation, formal analysis, writing – review and editing. **Katie McCarthy:** methodology, investigation, data curation, formal analysis, writing – review and editing. **Sophie Isobel:** conceptualisation, supervision, formal analysis, writing – original draft, investigation, methodology, writing – review and editing.

## Patient or Public Contribution

This study was designed, led and written in equal partnership between members of the team with lived, clinical and academic expertise. The work is based upon commitment to integration of lived expertise into health service provision and practice‐based evidence production.

## Conflicts of Interest

The authors declare no conflicts of interest.

## Data Availability

The authors have nothing to report.
